# *N*,*N*′-Bis[(*E*)-4-cyano­benzyl­idene]urea

**DOI:** 10.1107/S1600536809002244

**Published:** 2009-01-23

**Authors:** De-Hong Wu, Ling Hu

**Affiliations:** a Ordered Matter Science Research Center, College of Chemistry and Chemical Engineering, Southeast University, Nanjing 210096, People’s Republic of China

## Abstract

The mol­ecule of the title compound, C_17_H_10_N_4_O, has crystallographically imposed *C*_2_ symmetry. The urea group and the benzene ring are nearly coplanar, the dihedral angle between them being 4.15 (7)°. The crystal packing is stabilized by aromatic π–π stacking inter­actions, with a centroid-to-centroid separation of 3.833 (4) Å.

## Related literature

For a general background on the use of nitriles as starting materials, see: Íkizler & Sancak (1992 ▶). For the products of the condensation of urea with alkynes, see: Martínez-García *et al.* (2004 ▶).
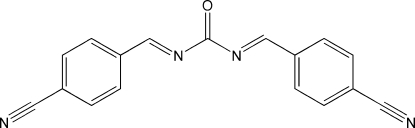


## Experimental

### 

#### Crystal data

C_17_H_10_N_4_O
*M* *_r_* = 286.29
Monoclinic, 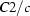

*a* = 10.552 (4) Å
*b* = 11.687 (5) Å
*c* = 12.198 (3) Å
β = 99.94 (4)°
*V* = 1481.7 (9) Å^3^
*Z* = 4
Mo *K*α radiation
μ = 0.08 mm^−1^
*T* = 291 (2) K
0.36 × 0.30 × 0.28 mm

#### Data collection

Rigaku Mercury2 diffractometer
Absorption correction: multi-scan (*CrystalClear*; Rigaku, 2005 ▶) *T*_min_ = 0.96, *T*_max_ = 0.98
6461 measured reflections
1423 independent reflections
1107 reflections with *I* > 2σ(*I*)
*R*_int_ = 0.041

#### Refinement

*R*[*F* ^2^ > 2σ(*F* ^2^)] = 0.072
*wR*(*F* ^2^) = 0.164
*S* = 1.06
1423 reflections
102 parameters
1 restraint
H-atom parameters constrained
Δρ_max_ = 0.43 e Å^−3^
Δρ_min_ = −0.27 e Å^−3^

### 

Data collection: *CrystalClear* (Rigaku, 2005 ▶); cell refinement: *CrystalClear*; data reduction: *CrystalClear*; program(s) used to solve structure: *SHELXS97* (Sheldrick, 2008 ▶); program(s) used to refine structure: *SHELXL97* (Sheldrick, 2008 ▶); molecular graphics: *SHELXTL* (Sheldrick, 2008 ▶); software used to prepare material for publication: *SHELXTL*.

## Supplementary Material

Crystal structure: contains datablocks I, global. DOI: 10.1107/S1600536809002244/rz2289sup1.cif

Structure factors: contains datablocks I. DOI: 10.1107/S1600536809002244/rz2289Isup2.hkl

Additional supplementary materials:  crystallographic information; 3D view; checkCIF report

## Figures and Tables

**Fig. 1.**
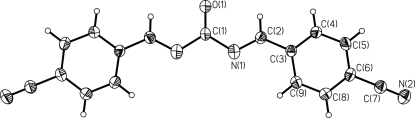
The molecular structure of the title compound, showing the atomic numbering scheme. Displacement ellipsoids are drawn at the 30% probability level. Unlabelled atoms are related to the labelled atoms by (-x, y, 1/2 - z).

## supplementary crystallographic information

### Comment

Nitriles are parent compounds for the preparation of various functional organic materials having imidazole, triazole, or thidiazole functionalities (Íkizler & Sancak, 1992). The Schiff-base compounds derived from the condensation of urea with alkynes are very few (Martínez-García *et al.*, 2004) because of the low reactivity of the NH_2_ group of urea. Here we report the crystal structure of the title compound, which was obtained by the reaction of urea with 4-cyanobenzaldehyde in acetic acid with ammonium chloride as a catalyzer.

The molecule of the title compound (Fig. 1) possesses a crystallographically imposed *C*_2_ symmetry, with atoms C1 and O1 located on a two-fold axis. The urea group and the aromatic rings are nearly coplanar, forming a dihedral angle of 4.15 (7)°. In the crystal packing, molecules are linked along the *a* axis by aromatic π–π stacking interactions, with centroid-to-centroid separations of 3.833 (4) Å, perpendicular interplanar distances of 3.474 (4) Å and centroid–centroid offsets of 1.620 (3) Å.

### Experimental

A mixture of 4-cyanobenzaldehyde (0.53 g, 4 mmol), urea (0.36 g, 6 mmol) and NH_4_Cl (0.10 g, 1.6 mmol) was heated with stirring at 100°C in 5 ml acetic acid for 5 h. After cooling, the reaction mixture was washed with cold water (3 × 50 ml) and the residue recrystallized from ethyl acetate/*n*-hexane (1:2 v/v) to afford the title compound (0.57 g, 50%). Single crystals suitable for X-ray structure analysis were obtained by the slow evaporation of an ethyl acetate solution in air.

### Refinement

All H atoms were placed in calculated positions and refined using a riding model aproximation, with C—H = 0.93 Å and with *U*_iso_(H) = 1.2*U*_eq_(C).

### Crystal data

**Table:**

C_17_H_10_N_4_O	*F*(000) = 592
*M**_r_* = 286.29	*D*_x_ = 1.283 Mg m^−^^3^
Monoclinic, *C*2/*c*	Mo *K*α radiation, λ = 0.71073 Å
Hall symbol: -C 2yc	Cell parameters from 1587 reflections
*a* = 10.552 (4) Å	θ = 2.6–27.4°
*b* = 11.687 (5) Å	µ = 0.08 mm^−^^1^
*c* = 12.198 (3) Å	*T* = 291 K
β = 99.94 (4)°	Block, yellow
*V* = 1481.7 (9) Å^3^	0.36 × 0.30 × 0.28 mm
*Z* = 4	

### Data collection

**Table:**

Rigaku Mercury2 diffractometer	1423 independent reflections
Radiation source: fine-focus sealed tube	1107 reflections with *I* > 2σ(*I*)
graphite	*R*_int_ = 0.041
Detector resolution: 13.6612 pixels mm^-1^	θ_max_ = 26.0°, θ_min_ = 2.9°
CCD profile fitting scans	*h* = −12→12
Absorption correction: multi-scan (*CrystalClear*; Rigaku, 2005)	*k* = −14→14
*T*_min_ = 0.96, *T*_max_ = 0.98	*l* = −15→15
6461 measured reflections	

### Refinement

**Table:**

Refinement on *F*^2^	Primary atom site location: structure-invariant direct methods
Least-squares matrix: full	Secondary atom site location: difference Fourier map
*R*[*F*^2^ > 2σ(*F*^2^)] = 0.072	Hydrogen site location: inferred from neighbouring sites
*wR*(*F*^2^) = 0.164	H-atom parameters constrained
*S* = 1.06	*w* = 1/[σ^2^(*F*_o_^2^) + (0.0535*P*)^2^ + 1.99*P*] where *P* = (*F*_o_^2^ + 2*F*_c_^2^)/3
1423 reflections	(Δ/σ)_max_ < 0.001
102 parameters	Δρ_max_ = 0.43 e Å^−^^3^
1 restraint	Δρ_min_ = −0.27 e Å^−^^3^

### Special details

**Table:**

Geometry. All e.s.d.'s (except the e.s.d. in the dihedral angle between two l.s. planes) are estimated using the full covariance matrix. The cell e.s.d.'s are taken into account individually in the estimation of e.s.d.'s in distances, angles and torsion angles; correlations between e.s.d.'s in cell parameters are only used when they are defined by crystal symmetry. An approximate (isotropic) treatment of cell e.s.d.'s is used for estimating e.s.d.'s involving l.s. planes.
Refinement. Refinement of *F*^2^ against ALL reflections. The weighted *R*-factor *wR* and goodness of fit *S* are based on *F*^2^, conventional *R*-factors *R* are based on *F*, with *F* set to zero for negative *F*^2^. The threshold expression of *F*^2^ > σ(*F*^2^) is used only for calculating *R*-factors(gt) *etc*. and is not relevant to the choice of reflections for refinement. *R*-factors based on *F*^2^ are statistically about twice as large as those based on *F*, and *R*- factors based on ALL data will be even larger.

### Fractional atomic coordinates and isotropic or equivalent isotropic displacement parameters (Å^2^)

**Table:**

	*x*	*y*	*z*	*U*_iso_*/*U*_eq_	
C1	0.0000	0.1727 (3)	0.2500	0.0459 (8)	
C2	0.0655 (2)	0.1864 (2)	0.45184 (18)	0.0476 (6)	
H2A	0.0645	0.1068	0.4491	0.057*	
C3	0.1028 (2)	0.2366 (2)	0.56187 (18)	0.0419 (6)	
C4	0.1265 (2)	0.1659 (2)	0.65419 (19)	0.0505 (6)	
H4A	0.1174	0.0872	0.6445	0.061*	
C5	0.1636 (2)	0.2094 (2)	0.7610 (2)	0.0526 (7)	
H5A	0.1790	0.1605	0.8220	0.063*	
C6	0.1773 (2)	0.3265 (2)	0.77534 (19)	0.0484 (6)	
C7	0.2135 (3)	0.3729 (2)	0.8858 (2)	0.0564 (7)	
C8	0.1546 (2)	0.3991 (2)	0.6841 (2)	0.0544 (7)	
H8A	0.1640	0.4777	0.6942	0.065*	
C9	0.1181 (2)	0.3551 (2)	0.5786 (2)	0.0525 (7)	
H9A	0.1034	0.4043	0.5179	0.063*	
N1	0.0325 (2)	0.2397 (2)	0.35441 (17)	0.0643 (7)	
N2	0.2416 (3)	0.4101 (2)	0.9729 (2)	0.0772 (8)	
O1	0.0000	0.0683 (2)	0.2500	0.0655 (8)	

### Atomic displacement parameters (Å^2^)

**Table:**

	*U*^11^	*U*^22^	*U*^33^	*U*^12^	*U*^13^	*U*^23^
C1	0.0458 (18)	0.055 (2)	0.0363 (17)	0.000	0.0039 (13)	0.000
C2	0.0485 (13)	0.0509 (14)	0.0428 (12)	−0.0062 (10)	0.0065 (10)	−0.0032 (9)
C3	0.0412 (12)	0.0466 (13)	0.0384 (12)	−0.0019 (9)	0.0081 (9)	0.0009 (9)
C4	0.0628 (15)	0.0472 (14)	0.0412 (13)	−0.0034 (11)	0.0085 (10)	−0.0011 (10)
C5	0.0608 (16)	0.0575 (16)	0.0394 (12)	0.0015 (12)	0.0087 (11)	0.0065 (11)
C6	0.0449 (13)	0.0595 (15)	0.0404 (13)	0.0016 (11)	0.0058 (10)	−0.0095 (11)
C7	0.0638 (16)	0.0577 (16)	0.0468 (15)	0.0041 (12)	0.0073 (12)	−0.0036 (12)
C8	0.0626 (16)	0.0484 (14)	0.0509 (14)	−0.0036 (11)	0.0063 (11)	−0.0025 (11)
C9	0.0604 (15)	0.0522 (14)	0.0435 (13)	−0.0021 (12)	0.0049 (11)	0.0055 (11)
N1	0.0696 (15)	0.0745 (17)	0.0472 (12)	0.0003 (12)	0.0055 (10)	0.0023 (10)
N2	0.106 (2)	0.0707 (17)	0.0511 (14)	0.0061 (14)	0.0034 (13)	−0.0151 (12)
O1	0.098 (2)	0.0506 (16)	0.0442 (14)	0.000	0.0009 (13)	0.000

### Geometric parameters (Å, °)

**Table:**

C1—O1	1.221 (4)	C4—H4A	0.9300
C1—N1	1.484 (3)	C5—C6	1.384 (4)
C1—N1^i^	1.484 (3)	C5—H5A	0.9300
C2—N1	1.334 (3)	C6—C8	1.387 (4)
C2—C3	1.455 (3)	C6—C7	1.442 (3)
C2—H2A	0.9300	C7—N2	1.138 (3)
C3—C4	1.384 (3)	C8—C9	1.377 (3)
C3—C9	1.405 (3)	C8—H8A	0.9300
C4—C5	1.390 (3)	C9—H9A	0.9300
			
O1—C1—N1	121.83 (16)	C6—C5—H5A	120.5
O1—C1—N1^i^	121.83 (16)	C4—C5—H5A	120.5
N1—C1—N1^i^	116.3 (3)	C5—C6—C8	120.2 (2)
N1—C2—C3	128.4 (2)	C5—C6—C7	119.7 (2)
N1—C2—H2A	115.8	C8—C6—C7	120.1 (2)
C3—C2—H2A	115.8	N2—C7—C6	179.5 (3)
C4—C3—C9	118.0 (2)	C9—C8—C6	120.2 (2)
C4—C3—C2	119.5 (2)	C9—C8—H8A	119.9
C9—C3—C2	122.5 (2)	C6—C8—H8A	119.9
C3—C4—C5	121.8 (2)	C8—C9—C3	120.7 (2)
C3—C4—H4A	119.1	C8—C9—H9A	119.7
C5—C4—H4A	119.1	C3—C9—H9A	119.7
C6—C5—C4	119.1 (2)	C2—N1—C1	120.3 (3)

Symmetry codes: (i) −*x*, *y*, −*z*+1/2.

## References

[bb1] Íkizler, A. A. & Sancak, K. (1992). *Monatsh. Chem.* **123**, 257–263.

[bb2] Martínez-García, A., Ortiz, M., Martínez, R., Ortiz, P. & Reguera, E. (2004). *Ind. Crops Prod.* **19**, 99–106.

[bb3] Rigaku (2005). *CrystalClear* Rigaku Corporation, Tokyo, Japan.

[bb4] Sheldrick, G. M. (2008). *Acta Cryst.* A**64**, 112–122. 10.1107/S0108767307043930 18156677

